# Impact of stroke on people that receive rehabilitation and are living in Ankara, Turkey

**DOI:** 10.1177/22799036231204321

**Published:** 2023-10-08

**Authors:** Orkun Tahir Aran, Barkın Köse, Gunilla Erikson, Susanne Guidetti

**Affiliations:** 1 Department of Occupational Therapy, Faculty of Health Sciences, Hacettepe University, Ankara, Turkey; 2 Occupational Therapy Department, Gülhane Faculty of Health Sciences, University of Health Sciences, Ankara, Turkey; 3 Division of Occupational Therapy, Department of Neurobiology, Care Sciences and Society, Karolinska Institutet, Huddinge, Sweden; 4 Theme Women’s Health and Allied Health Professionals, Medical Unit Occupational Therapy and Physiotherapy, Karolinska University Hospital, Huddinge, Sweden

**Keywords:** Stroke impact scale, stroke, occupational therapy, disability, rehabilitation, Turkey, activities of daily living

## Abstract

**Background:**

Clinical guidelines for stroke rehabilitation and practices vary between high and low/middle-income countries (LMICs). Knowledge of the perceived impact of stroke in Turkey is limited. Understanding these perceptions can serve as a basis for developing rehabilitation.

**Design and methods:**

The aim was to investigate and compare the perceived impact of stroke in two groups of people living in Ankara. A cross-sectional study with 150 participants divided by stroke onset (Group I: stroke onset <12 months; Group II: >12 months) was conducted. The Barthel Index was used to describe the level of independence in daily living activities and stroke severity. The Stroke Impact Scale (SIS 3.0) was used to investigate the perceived impact of stroke.

**Results:**

The proportion of mild strokes was 78 and 82%, respectively, and 46% of participants in the total sample were moderately dependent. The impact of stroke was high; mean domain scores were below 50 in six of the eight SIS domains.

**Conclusions:**

Turkish stroke survivors perceived a higher impact of stroke regardless of the time passed since stroke onset, compared to survivors from other countries, including other LMICs. The high impact among survivors with mostly mild stroke indicates that Turkish survivors might not receive adequate rehabilitation. The content of rehabilitation services needs to be developed, and an evaluation of individually tailored interventions, preferably with a multidisciplinary approach, is warranted to find ways to decrease the perceived impact of stroke among Turkish stroke survivors.

## Background

With approximately 16 million incidences annually and 62 million survivors living with consequences, stroke is one of the main causes of chronic disability worldwide.^1^ Stroke causes disability that affects an individual in many ways, such as decreased motor and cognitive functions, daily functioning, and social participation.^2,3^ The majority of stroke survivors continue their daily lives by being dependent on others and facing limitations in social and leisure participation.^4^

According to the Global Burden of Disease, global incidence of stroke increased by 76% in 2017 (11.9 million events) but the age-standardized global stroke incidence rate (i.e., new stroke incidents per 100,000 people) decreased overall by 11% (150.5 per 100,000).^5^ In addition, there was an increase in stroke prevalence of 95 and 3% in age-standardized rates. Number of incidences was highest in upper-middle income countries and lowest in high-income counties (HIC), whereas the prevalence of stroke was highest in upper-middle income countries and lowest in the low-income countries (LIC).^6^

After a stroke, there is often a need for rehabilitation. Clinical guidelines regarding rehabilitation and content of rehabilitation varies between countries, as do the practices.^7^ Most guidelines recommend inpatient rehabilitation for people with moderate and severe stroke while few reported guidelines for discharge and outpatient rehabilitation/community-based rehabilitation. Only HIC had available data on access to inpatient rehabilitation. Information on access to rehabilitation services was reported to be scarce or inaccessible in LIC.^7^

Turkey is a lower-middle income country (LMIC)^8^ but the data on prevalence of stroke is unknown.^9^ According to Turkish health statistics, 1% of the population had a stroke in 2017 and stroke was the third-leading cause of death (23.8 per 100,000 people).^10^ However, these numbers could be underestimated and might be higher.^11^ Further information on proportions of people with stroke who have had access to inpatient rehabilitation appears to be unknown.^7^

To understand and be able to portray a larger picture of the consequences of a stroke, the perceptions of those having had stroke is essential. The need for and the impact of rehabilitation needs to be studied and compared between different healthcare systems with varying rehabilitation guidelines and efforts. There is an uncertainty regarding how to best provide rehabilitation interventions for people with disabilities after stroke in LMIC as most research emanates from HIC,^12^ as this knowledge needs to be understood and translated to its new societal context. Currently, in Turkey, research regarding rehabilitation after stroke is sparse and there is a knowledge gap regarding the situation after rehabilitation of people living with the consequences after stroke. Understanding how everyday life is perceived by people with stroke and their family members is the basis/foundation for providing a goal-oriented/based rehabilitation.^13^ Our intention with this study is to present information about rehabilitation after a stroke in Turkey as well as an awareness of people’s perceived impact of stroke in Turkey compared to other countries. This knowledge can provide support for developing stroke rehabilitation further both in Turkey and in other LMIC countries based on their healthcare systems.

### Rehabilitation structure in Turkey

In Turkey there are two types of public institutions providing medical and rehabilitation services: state hospitals and university hospitals. There are also private hospitals providing these services.^11^ Institutional organizations that offer stroke care focus on reducing mortality and morbidity. However, in recent years, significant action plans and improvements have been made, such as opening accredited stroke hospitals, and taking precautionary measures to decrease risk factors (cigarette ban, etc.). Medical and rehabilitation services are covered by the Turkish Republic social security act called the Health Application Communiqué (HAC).^14^ The HAC specifies that the Social Security Institution must cover 30 sessions of rehabilitation per individual over a 1-year period. In addition, if a medical council reports that the patient needs to have additional sessions for recovery, HAC covers 30 more sessions.

The rehabilitation structure in Turkey is defined in the HAC and covers rehabilitation in package programs such as neurorehabilitation, orthopedic rehabilitation, and so on.^14^ A package program includes 30 sessions of rehabilitation, and it incorporates physiotherapy (majority of the package), speech therapy, audiology, and occupational therapy. The acute phase of the rehabilitation is carried out in an inpatient clinic where individuals usually receive physical therapy.

In Turkey, stroke survivors and impact of stroke on health-related quality of life and independence in daily activities have been investigated. These studies indicate a perceived lower quality of life for people after stroke than healthy subjects and that their independence in functioning, including home and work activities, have decreased.^15–17^ However, in-depth analysis is required of people’s own perceived impact of stroke to better meet their needs for rehabilitation. The perceived impact of stroke has been analyzed in one cross-sectional study in Turkey more than a decade ago, using the 16-item Stroke Impact Scale (SIS)^18^ including items concerning physical functions. No detailed analysis was performed of the perceived impact of stroke among the individuals in the small sample, who were recruited more than 3 months after stroke. Only the physical domains were investigated, thereby excluding perceptions of more hidden stroke impact as memory, participation, emotions, and so on. Stroke research is warranted in LMIC^12^ and as WHO emphasizes, information regarding the need for rehabilitation services based on how people perceive the impact of stroke and everyday functioning in the acute/subacute/chronic phase needs to be understood.^19^ The understanding generated can be a basis for developing a more individualized and goal-oriented rehabilitation and/or a remodeling of the rehabilitation services provided. Therefore, as a starting point for developing this knowledge the aim of this study was to investigate and compare the perceived impact of stroke in two groups of people living in Ankara who had a stroke at different time points and have received rehabilitation.

## Methods

### Design

A cross-sectional comparative design was used involving a conventional sample divided into two groups (Group I: stroke onset <12 months; Group II: >12 months) receiving outpatient rehabilitation after stroke. The Non-interventional Clinical Research Ethics Board of Hacettepe University approved this study (GO 19/520).

### Participants

**Figure 1.**
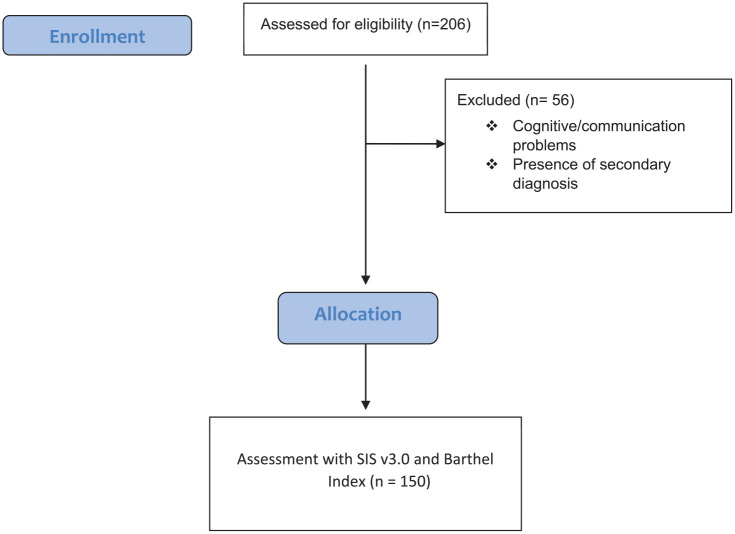
Participant allocation.

All 150 participants (95 male, 55 female) were recruited from the Occupational Therapy Department, Faculty of Health Sciences, Hacettepe University, between January and September 2019. Inclusion criteria were 1) having a stroke diagnosis, 2) having a score of >23 on the Mini-Mental State Exam (MMSE)^20^ and, 3) being able to understand and answer the questions (Figure 1). After all eligible patients were informed about the design and purpose of the study, signed informed consent was obtained. For participants who couldn’t read the informed consent form, the document was orally presented to them or their legally authorized representative, in the presence of an independent witness and hence the informed consent was obtained from illiterate participants as well.

### Data collection

All participants were assessed while receiving rehabilitation as outpatients. Demographic assessment forms—including age, gender, time passed since the stroke, dominant upper extremity, side of stroke, educational background, marital status, and income source—were created by the two first authors (OTA, BK). The second author (BK) gathered demographic information via face-to-face interviews with help from an occupational therapy intern. Data collection of the assessments was administered by the second author (BK). The participants were divided into two samples based on time for stroke onset as being Group I (had a stroke in 0–12 months), or Group II (had a stroke for more than 12 months ago).

### Assessments

The Stroke Impact Scale 3.0^21^ was used to investigate the perceived impact of stroke. The Barthel Index^22^ was used to describe the level of independence and to categorize the severity of stroke in the sample.

#### Stroke Impact Scale 3.0 (SIS)

The SIS 3.0 consists of eight domains and 59 questions to assess the patient’s perception of quality of life after a stroke, and the questions apply to the previous four weeks. The eight domains are Strength, Hand function, Activities of daily living / Instrumental activities of daily living (ADL/IADL), Mobility, Communication, Emotion, Memory and thinking, and Participation. Each question is scored by evaluating the difficulty experienced in the last week on a five-point Likert scale. In addition to the domains, SIS 3.0 includes an assessment of post-stroke recovery with a visual analog scale of 0–100 (0: no improvement, 100: complete recovery). The domain scores (range 0–100) are calculated by the following formula: ([Actual raw score − lowest possible raw score]/possible raw score) × 100 (22). The Turkish version of SIS 3.0 has been tested and demonstrated high validity and reliability with Cronbach’s alpha score ranging from 0.96 to 0.99 and the ICC score ranging from 0.95 to 0.99.^23^

#### Barthel Index of Activities of Daily Living (BI)

The BI evaluates the level of independence in ADL in 10 self-care and mobility activities^22^ with a range from 0 to 100—a lower score indicating greater dependency. The classification of BI scores are as follows; BI < 20 total dependent, BI = 20–60: severely dependent, BI = 61–90: moderately dependent, and BI = 91–99: slightly dependent, BI = 100: independent.^22^ Stroke severity was also categorized based on the BI scores: severe stroke, BI ≤ 15; moderate stroke, BI = 15–49; and mild stroke BI = 50–100.^24^ The Turkish validity and reliability of the BI^25^ showed Cronbach’s alpha score of 0.93 and a 0.77 ICC score for stroke.

### Statistical analysis

IBM SPSS v.23 for Windows was used to analyze the data. Demographic results were descriptive and expressed as percentages, means, standard deviation, or medians. Independent samples *t*-test was used to compare the SIS scores between the groups. Additionally, the SIS scores were analyzed in relation to gender, educational levels (university and tertiary school compared to lower education levels), and working status (actively working compared to non-working participants) with independent samples *t*-test. For both groups, multiple regression analyses were compiled to determine the effects of age, gender, education, BI dependency (hence will be named as ADL dependency), and stroke severity on the perceived impact of stroke.

## Results

The participants’ demographic characteristics are presented in Table 1. The proportion of men was higher in Group I (70% compared to 50%). The proportion of persons not graduated from high school in Groups I and II was 67 and 88%, and the proportion of retired were 50 and 70% respectively. The clinical characteristics of the sample and the perceived impact of stroke are presented in Table 2. The majority in both groups had had a mild stroke; 78 and 82% respectively. Most of the participants in both groups were “moderately dependent” according to the BI (46%).

**Table 1.** Socio-demographics of the participants (*n* = 150).

Participants with stroke	Group I (*n* = 100) (0–12 months)			Group II (*n* = 50) (>12 months)		
	*n*	SD	%	*n*	SD	%
Age (years)						
Mean	59.84	8.14		61.10	9.46	
Gender						
Male	70	–	70	25	–	50
Female	30	–	30	25	–	50
Number of children						
Mean	2.09	1.18	–	2.80	1.57	–
Dominant extremity (hand)						
Left	13	–	13	6	–	7
Right	87	–	87	44	–	43
Affected extremity						
Left	54	–	54	24	–	48
Right	46	–	46	26	–	52
Level of education						
No education	11	–	11	5	–	10
Primary school	17	–	17	18	–	36
Secondary school	39	–	39	21	–	42
Tertiary school	15	–	15	6	–	12
University	18	–	18	–	–	
Housing						
Own house	63	–	63	28	–	56
Rent	32	–	32	18	–	36
Living with relative	5	–	5	4	–	8
Marital status						
Married	89	–	89	43	–	86
Single/widowed	11	–	11	7	–	14
Use of assistive aids after stroke						
Yes	26	–	26	9	–	18
No	74	–	74	41	–	82
Source of income						
No income	5	–	5	7	–	14
Employed in private sector	20	–	20	7	–	14
Employed in government	17	–	13	–	–	–
Retired	56	–	56	35	–	70
Disability aid	2	–	2	1	–	2

**Table 2.** Clinical characteristics of participants and perceived impact of stroke.

Participants with stroke	Group 1 (*n* = 100) (0–12 months)			Group II (*n* = 50) (>12 months)			*p*-Value
	*n*	SD	%	*n*	SD	%	
Dependency by Barthel Index							
Dependent (0–59)	26	–	26	9	–	18	–
Moderately dependent (60–90)	46	–	46	23	–	46	–
Independent (91–100)	28	–	28	18	–	36	–
Stroke severity according to BI							
Severe stroke; <15	3		3	5	–	10	
Moderate stroke; 15–49	19		19	4	–	8	
Mild stroke; 50–100	78		78	41	–	82	
SIS 3.0 domains	Mean	SD		Mean	SD		
Strength	36.18	28.32		37.87	29.35		0.63
Memory	66.39	29.35		66.28	28.79		0.49
Emotion	47.56	12.32		43.11	16.22		0.032*
Communication	69.19	30.93		69.64	31.79		0.53
ADL/IADL	40.55	20.08		36.50	26.31		0.14
Mobility	45.61	24.96		44.44	29.45		0.40
Hand function	29.70	28.43		17.90	20.80		0.005*
Participation	41.47	24.61		35.11	31.36		0.09
Recovery	32.15	25.16		32.30	26.12		0.51

SIS: Stroke Impact Scale 3.0; *p*: One-way ANOVA test.

* *p* < 0.05.

The lowest scores on the SIS (indicating more difficulties) in both Group I and II were in the domain Hand function, with a mean score of 29.7 and 17.19, respectively. The highest score (indicating less difficulties) in both groups was in the domain of Communication, with a mean score of 69.19 and 69.64, respectively. There were few significant differences between the groups in the SIS domain scores. Group I had significantly higher scores in the domains Emotion (*p* = 0.032) and Hand function (*p* = 0.005) (Table 2).

Additional analyses were conducted to compare SIS domains in the total sample in relation to gender, educational levels and working status (Table 3). Men had statistically significant higher scores in the Memory domain (*p* = 0.003) than women. Education levels were grouped into high school versus not graduating from high school (i.e., no education, primary and secondary school). Participants with higher educational levels had significantly higher scores on the Strength (*p* = 0.002) and Hand function (*p* = 0.009) domains. Lastly, the domain scores were analyzed according to working status. The ones with no income, disability aid or pension constituted one group and were compared to the ones working. The participants that worked had significantly higher scores in the domains Strength (*p* = 0.0001) and ADL (*p* = 0.024).

**Table 3.** Comparisons of impact of stroke in relation to gender, education levels, and working status.

SIS 3.0 domains	Groups				*p*
	Male	*n* = 95	Female	*n* = 55	
	Mean	SD	Mean	SD	
Strength	38.81	29.86	33.18	26.10	0.42
Memory	67.63	23.10	63.63	31.87	0.003*
Emotion	45.88	14.21	46.41	13.33	0.90
Communication	70.13	31.12	67.98	31.35	0.90
ADL/IADL	38.76	20.95	39.95	24.75	0.90
Mobility	45.88	25.73	44.09	27.84	0.08
Hand function	25.05	27.73	27.00	25.73	0.63
Participation	41.91	26.67	35.02	27.81	0.57
Recovery	40.29	19.88	47.33	19.02	0.46
SIS 3.0 domains	Education—below high school *n* = 111		Education—high school and above *n* = 39		*p*
	Mean	SD	Mean	SD	
Strength	32.26	25.09	49.51	33.97	0.002*
Memory	65.81	28.33	67.84	21.29	0.047*
Emotion	46.72	14.31	44.25	12.44	0.56
Communication	69.90	30.43	67.76	33.35	0.10
ADL/IADL	39.14	23.30	39.35	19.62	0.19
Mobility	45.15	27.09	45.42	24.88	0.75
Hand function	27.13	28.57	21.13	20.07	0.009*
Participation	36.72	27.36	46.93	25.59	0.08
Recovery	43.00	19.07	43.43	21.71	0.31
SIS 3.0 domains	Not-working *n* = 109		Working *n* = 41		*p*
	Mean	SD	Mean	SD	
Strength	32.91	23.90	46.95	36.79	0.0001*
Memory	69.11	26.47	58.96	25.92	0.60
Emotion	47.02	14.19	43.58	12.73	0.59
Communication	71.21	31.88	64.37	28.87	0.58
ADL/IADL	39.83	23.87	37.50	17.78	0.024*
Mobility	45.80	36.56	43.68	26.40	0.45
Hand function	26.53	28.03	23.75	22.79	0.15
Participation	36.82	27.61	46.17	25.16	0.052
Recovery	44.05	19.36	40.90	20.82	0.56

SD: Standard Deviation; SIS: Stroke Impact Scale; *p*: Independent samples *T* test.

* *p* < 0.05.

Multiple linear regression models were compiled to investigate the effects of age, gender, education, ADL dependency and Stroke severity (according to BI scores) on each SIS domain (Table 4). For Group I, most of the domains were affected by stroke severity and ADL dependency, while education and age affected some of the domains. The regression analyses showed that 20% of SIS Strength domain, 38% of the SIS ADL domain, and 19% of the SIS domain Hand function were explained by education and stroke severity, ADL dependency and age, ADL dependency respectively. For Group II, nearly all the domains were affected by stroke severity and ADL dependency. The analyses showed that 70% of ADL domain, 40% of Mobility, and 37% of Hand function domains were explained by ADL dependency, Stroke severity, and Stroke severity respectively.

**Table 4.** Multiple linear regression results (all features have significant *p* values (*p* < 0.05).

Dependent variable	Groups	Models	*F*	SD	*t*	*R* ^2^
SIS-strength	I	1. Education	16.21	3.56	4.02	0.14
		2. Education, stroke severity	13.92	5.57	3.00	0.21
	II	1. Stroke severity	9.21	5.38	3.03	0.16
SIS-memory	I	1. Stroke severity	6.11	3.44	2.46	0.06
	II	1. Stroke severity	12.47	5.07	3.95	0.20
		2. Education	8.66	11.12	2.01	0.27
SIS-emotion	I	1. Education	7.07	2.54	–2.60	0.07
SIS-communication	I	1. Stroke severity	10.47	4.03	3.41	0.01
		2. Education	7.70	6.37	−2.15	0.13
		3. ADL dependency	6.67	6.51	−2.02	0.17
	II	1. ADL dependency	12.54	3.28	3.54	0.20
		2. Education	11.54	11.80	2.92	0.33
SIS-ADL	I	1. ADL dependency	50.65	1.52	6.57	0.34
		2. Age	29.47	0.19	–0.19	0.38
	II	1. ADL dependency	116.92	1.64	10.81	0.70
SIS-mobility	I	1. ADL dependency	12.42	4.21	3.95	0.19
	II	1. Stroke severity	32.56	4.53	5.70	0.40
SIS-hand	I	1. ADL, dependency	22.58	2.12	4.73	0.19
	II	1. Stroke severity	27.72	3.32	5.26	0.37
SIS-participation	I	1. Stroke severity	19.87	3.06	4.45	0.17
	II	1. Education	12.72	11.86	4.17	0.21
		2. Stroke severity	10.44	5.41	2.58	0.31
SIS-recovery	I	1. Stroke severity	10.22	2.26	3.19	0.01

ADL: Activities of Daily Living, stroke severity: Barthel Index Stroke Severity classification; ADL dependency: Barthel Index regular scoring based classification; SIS: Stroke Impact Scale.

## Discussion

Turkey is a LMIC^8^ where stroke is one of the leading causes of mortality and disability^26^ but there is limited information about the perceived impact of stroke. This is the first study to analyze the perceived impact of stroke at different time periods after stroke. The perceived impact of stroke was reasonably similar between the groups, despite that time since stroke onset differed. The groups had mean domain scores in SIS below 50 in six of the eight domains which indicate that the sample in general perceived many difficulties in performing activities and in participation in everyday life. These quite low SIS mean domain scores in the study sample are lower than what other samples in different countries have reported for individuals at differing time points since stroke onset. These differences will be further discussed and in relation to the implications for rehabilitation. The mean age in the study sample were like the average age for people with stroke in Turkey. There were however more females participating, which is not in line with Turkish data on gender distribution after stroke. Most of the participants had had a mild stroke and were moderately dependent according to BI.

Both groups perceived the domain Hand function badly impacted (SIS mean scores 29.7 and 17.9 respectively) by their stroke. Additionally, the Strength (Group I), Participation, and ADL/IADL domains (Group II) were the highest impacted domains (more difficulties) with mean scores below 40. Low scores in the same domains were also found in studies from the US^21,27^ Sweden,^28,29^ Uganda,^30,31^ and Brazil.^32^ However, in the present study, the mean scores in these domains were much lower in general and even lower in comparison to the Turkish SIS validity study,^23^ with exceptions for Strength and Mobility domains.

The Stroke recovery mean scores (i.e., 32) were very low in both groups and much lower compared to the Swedish,^30,31^ Brazilian,^32^ and American^21^ studies. Duncan et al.^21^ found that the physical aspects of stroke, as well as the Emotion and Participation domain scores, predict the perceptions of Stroke recovery. This finding is in line with the results in the present study where low mean scores were found in the Hand function, ADL/IADL, Strength, and Emotion domains, that is, Stroke recovery is accepted as a function of the domain scores^17^ also in the Turkish context.

For both groups the regression analyses indicated that the perceived consequences of stroke were explained by ADL dependency and stroke severity levels. In addition to that, the Group I had slightly higher mean scores in almost all domains and had significantly higher scores in the Emotion and Hand function domains than Group II. However, the reported scores indicate that both groups perceived problems in the performance of activities and in their participation. A possible reason for the perceived lower impact in the earlier phases after stroke in the Emotion and Hand function domains might be based on the participants’ lack of awareness of the consequences of stroke. During the first months after a stroke, a survivor might not yet have faced so many limitations in their daily life and will therefore not find struggles as severe as in the subsequent phases. On the other hand, the number of rehabilitation professionals working in stroke rehabilitation in Turkey is however insufficient (except for physiotherapists). Only one service is covered by HAC which means that an individual is not allowed to get both occupational therapy and physiotherapy in the same rehabilitation package. The individual gets 30 sessions of inpatient rehabilitation (up to 60 sessions if the hospital’s medical council allows). When an individual receives rehabilitation within the scope of only one profession, interdisciplinary teamwork will be disrupted. Following discharge, the individual cannot receive any rehabilitation services for 1 year unless they are willing to pay for services themselves. Therefore, it was thought that the structure of rehabilitation programs in Turkey might lead patients that receive rehabilitation within the first year after stroke onset to attain better functional outcomes because they get a more intense rehabilitation than patients in the later phases.

The socio-cultural and economic levels of the participants in the study sample might also have affected the SIS scores. Most of them lived on a pension or without income and the group of participants not working rated significantly more difficulties on Strength and ADL (see Table 3) than those working. Previous studies have shown that high inflation rates in Turkey affect citizens’ quality of life and future expectations and increase an individual’s economic responsibilities.^33,34^ Turkish Statistical Institute data state that the employment rate of people who have completed their education at a level lower than tertiary school was 50% and these individuals generally work at a minimum wage. In the present study, 74% of the participants had an education lower than tertiary school and this group perceived significantly more difficulties in Strength and Hand functions. Additionally, the Organization for Economic Co-operation and Development (OECD) report, “The Better Life Index,” and its analysis by Nar et al. state that the quality of life in Turkey is lower than in other OECD countries.^35,36^ While the quality of life of Turkish citizens is low,^35^ having a stroke might also decrease the quality of life, because of additional disabilities. As in the Ugandan sample,^30,31^ Turkish survivors certainly want and need to return to their former lives, continue working if they are of working ages and take care of their responsibilities to sustain themselves socio-economically.

The participants in the study sample reported perceived problems in Communication and Memory and thinking albeit fewer than in the other domains. Stroke survivors receiving rehabilitation in Turkey seem to be those with noticeable consequences, while those with more concealed consequences, such as memory problems or other cognitive impairment, seem not to be referred for rehabilitation to the same extent. In LMIC it appears to be less common than in HIC to include guidelines for stroke rehabilitation regarding cognitive rehabilitation^37^ although rehabilitation has been found effective.^37^ If people with mainly cognitive impairment after stroke and aphasia were referred to rehabilitation in the study context the results might have been different. Likewise, the current rehabilitation structure in Turkey, with focus mostly on motor impairment, might be affecting the perceived impact of stroke. More focus on ADLs,^37^ and home-based rehabilitation^38^ which has been shown to be effective might lessen the impact of stroke. These rehabilitation areas, however, seem not to be present in many of the guidelines in LMIC.^37^

It is known that poor and short-term access to rehabilitation services in LMIC affects stroke recovery and the burden of the stroke.^7^ Bernardt et al. stated that poor understanding of clinical leadership, absence of rehabilitation standards, insufficiency of the skilled workforce and availability of and access to skilled services as well as inadequate financial support in LMIC are the main obstacles to receiving effective stroke rehabilitation services.^39^ Collaborations between HIC and LMIC, however, are initiated to find ways to implement new models for rehabilitation services and education of staff^37^ as well as support for research efforts in LMIC. Collaboration between rehabilitation specialists, general practitioners/community healthcare and family caregivers in task-sharing to increase access to rehabilitation has been tested and found feasible but warrants further research.^12^

## Methodological considerations

This is the first study analyzing the perceived consequences of stroke in a sample receiving rehabilitation in Turkey. Although there are studies using similar scales in the literature in the Turkish context, this study is the first to examine the perceived impact of stroke in detail in a sample with mostly mild stroke in different phases. There are limitations such as lack of demographic data, that is, type of stroke, affected brain area, and monthly salary. Additionally, information regarding social security coverage (private, governmental, etc.), expectations in life and their daily responsibilities could have supplemented the findings. Even though a larger sample should have been preferred due to the prevalence of stroke in Turkey, this study provides important knowledge on the everyday life situations and the need for rehabilitation for people with stroke living in urban areas in Turkey. The sample size, the inclusion of participants only from urban areas and one rehabilitation setting, excluding patients with cognitive and communication problems and including only patients who were referred to occupational therapy limits the generalizability of the results to all Turkish stroke survivors. In addition to these, in this study, the SIS results were compared with scores from other countries ranging LIC to HIC. Although there is a study from Turkey among these studies, it was thought that our findings cannot be generalized for whole Turkey and other LMIC’s but can be used to point out the differences as a starting point. One other limitation is that the perceptions on impact of stroke were collected during the rehabilitation period. The participants’ perceptions might have been influenced by being in active training at this time point anticipating further progress.

## Conclusion

This study used SIS 3.0 to describe more in-depth how it was perceived to live with the consequences of stroke in a LMIC that is Turkey. The findings showed that the impacted domains were similar to those found in research from other countries but indicated more difficulties in the performance of activities and participation in everyday life. The results raise the question of whether the rehabilitation system in Turkey meets the needs of the survivors. The high perceived impact of stroke reported by individuals with mostly mild stroke emphasizes the need to develop the content in the rehabilitation programs further. Consequently, it is evident that the rehabilitation process can benefit from having a more multidisciplinary approach, having a greater focus on concealed consequences of stroke as well as targeting the interventions according to the individuals’ perceived needs. As the socio-economic situation seems to have implications for people living with the consequences of stroke in Turkey, this needs to be investigated further to get a better understanding of the impact on stroke survivors. This is surely relevant in many LMIC but the results regarding the perceived impact of stroke can nevertheless provide support in the development or remodeling of the rehabilitation services in other LMIC.

## Abbreviation

HIC: high-income countries

LIC: low-income countries

LMIC: lower-middle income countries

HAC: Health Application Communiqué

SIS: Stroke Impact Scale

MMSE: Mini-Mental State Exam

ADL: Activities of daily living

IADL: Instrumental activities of daily living

BI: Barthel Index of Activities of Daily Living
